# Behaviour of *Sotalia guianensis* (van Bénéden, 1864) (Cetacea, Delphinidae) and ethnoecological knowledge of artisanal fishermen from Canavieiras, Bahia, Brazil

**DOI:** 10.1186/1746-4269-8-18

**Published:** 2012-05-14

**Authors:** Martha Eloy Bandeira Costa, Yvonnick Le Pendu, Eraldo Medeiros Costa Neto

**Affiliations:** 1 Aquatic Mammals Research Group of Ilhéus (Grupo de Pesquisa em Mamíferos Aquáticos de Ilhéus), Department of Biological Sciences, State University of Santa Cruz (Universidade Estadual de Santa Cruz), 45662-900, Ilhéus, Bahia, Brazil; 2 Department of Biology, State University of Feira de Santana (Universidade Estadual de Feira de Santana), 44036-900, Feira de Santana, Bahia, Brazil

**Keywords:** Guiana dolphin, Behaviour patterns, Ethnobiology, Traditional knowledge

## Abstract

Artisanal fishermen, because of their direct and frequent contact with the aquatic environment, possess a wealth of knowledge about the natural history of the fauna of the region in which they live. This knowledge, both practical and theoretical, has been frequently utilized and integrated into academic research. Taking this into consideration, this study discusses the ethnoecological knowledge of artisanal fishermen from a community in Canavieiras, state of Bahia, Brazil regarding the Guiana dolphin (*Sotalia guianensis*), a typically costal member of the family Delphinidae that is little studied in this region. To this end, the behaviour of *S. guianensis* in Canavieiras was recorded over one year and the data obtained were compared with fishermen’s reports. A total of 609 hours of behavioural observations of *S. guianensis* was conducted from a fixed point in alternate morning and afternoon sessions between October 2009 and September 2010. Observations were conducted from a pier (15°40’59”S and 38°56’38”W) situated on the banks of the Pardo River estuary - the region’s main river - at 5.5 m above water level. For ethnoecological data collection, semi-structured interviews were carried out with 26 fishermen in May, June and September 2010 and January 2011 in the fishing community of Atalaia. Occasional boat expeditions were made with the fishermen to compare their reports with direct observations of the behaviour of *S. guianensis*. The results demonstrate that fishermen possess a body of knowledge about *S. guianensis* that describes in detail the main behavioural aspects of the species. They reported the presence of *S. guianensis* in the Pardo River estuary throughout the year and its gregarious behaviour. They cited a relationship between the movement of dolphins and tidal cycles, and their presence in the estuary associated with the search for food. In addition, the fishermen reported that numbers of infants in groups were proportional to group size. Behaviours described were compatible with the observations made *in situ* and with data found in the scientific literature, confirming the importance of traditional knowledge in complementing scientific data. One behaviour mentioned by the fishermen that had no equivalence in the scientific literature was confirmed *in situ* and, therefore, constitutes the first record for this species.

## Introduction

Artisanal fishermen maintain a direct and constant contact with the aquatic environment and need to understand the ecology and behaviour of the organisms they exploit in order to satisfactorily enact their fishing strategies [1,2]. Through observation and practice, they consequently develop a body of knowledge about the fauna of the region in which they live and this includes knowledge about non-target species [3]. This knowledge is frequently complemented by information transmitted orally over generations in the community within which they live [4]. The practical and theoretical knowledge presented by artisanal fishermen has been used by and integrated into academic research [5-7]; popular knowledge may thus support and complement scientific knowledge [8].

In general terms, sheltered and restricted areas of Central and South America, such as estuaries and bays, frequently include the presence of the *Sotalia guianensis* (van Bénéden, 1864), a small member of the family Delphinidae with coastal habits, whose distribution ranges from Honduras in Central America [9,10] to the state of Santa Catarina in Brazil [11]. This region has ecological systems with high productivity that are economically important where the artisanal fishermen exploit them through fishing; the fishing regularly takes place in the presence of *S. guianensis*[12]. This constant contact enables the fishermen to come to know ecological and behavioural aspects of the species [13].

Studies related to the knowledge of artisanal fishermen about *S. guianensis* behaviour remain scarce, despite the fact that this knowledge provides a source of information that may support processes for the sustainability, handling and conservation of the species [3,12]. Information obtained from fishermen may also lead to new studies to fill knowledge gaps about the biology and ecology of the fauna associated with fishing, whilst encouraging the involvement of fishermen in research activities can also be a means of supporting local culture [14].

In the Brazilian state of Bahia, behavioural studies about *S. guianensis* are concentrated in restricted areas, such as the estuaries of the Paraguaçu [15], Caravelas [16] and Cachoeira [17] rivers, while information about the species is scarce in other regions, as it is in municipality of Canavieiras, where the present study was carried out. Due to a lack of adequate data on abundance, non-natural mortality rates and other relevant parameters, *S. guianensis* is listed as “Data Deficient” in the International Union for Conservation of Nature and Natural Resources (IUCN) red list of threatened species [18].

The aim of this work was to describe various aspects of the behaviour of *S. guianensis* in the Pardo River estuary, which is situated in the municipality of Canavieiras, state of Bahia, and to compare obtained data with the ethnoecological knowledge of fishermen from a local artisanal fishing community. The study may support future initiatives aimed at the conservation of the *S. guianensis* in Canavieiras based on the local socio-cultural context.

### Study area

The municipality of Canavieiras is situated on the southern coast of the state of Bahia, 551 km from its capital Salvador, in northeastern Brazil (15°40’ S, 38°56’ W). It covers an area of 1,326 km^2^, with a coast of approximately 50 km and a resident population of 32,336 inhabitants. It has a humid to sub-humid climate; an average temperature of 25°C and its vegetation is made up of mangrove and restinga [19,20].

The Pardo River is significant amongst the rivers of Canavieiras and its basin covers an area of 30,360 km^2^. The Pardo River has one of the highest flows of river water discharge (about 3 km^3^ year^-1^), compared to other rivers in the southern region of the state of Bahia (e.g. Contas, Una, Almada and Cachoeira rivers). Furthermore, it has one of the greatest discharges of sediment on the coast, contributing mainly with suspended particulate matter (≈0.12 × 10^6^ T.year^-1^) [21].

The fishing communities of Canavieiras are made up of six main settlements: Oiticica, Puxim do Sul, Puxim de Fora, Barra Velha, Atalaia and Campinhos. These traditional communities are located along riverbanks and on islands. Together they constitute one of the municipality’s main economic sectors. The historical artisanal fishing community of Atalaia currently has a specialized work force that uses the water system of the Pardo River estuary and the marine coast to capture fish.

The Atalaia fishing community is made up of approximately 40 artisanal fishermen living with their families. This community was selected for the collection of ethnoecological data due to (i) its proximity to the municipal centre and the Pardo River estuary, (ii) its accessibility, (iii) the fact that it is a community that maintains traditional society characteristics and, above all, (iv) the frequent contact maintained by the fishermen with *S. guianensis* during their fishing activities.

## Methods

### Interviews with artisanal fishermen

The collection of ethnoecological data in the fishing community of Atalaia took place in May, June and September 2010 and January 2011 through semi-structured interviews with 26 fishermen, all of whom were male. Ages of those interviewed ranged from 17 to 62 years. Interviews were based on a previously prepared questionnaire containing questions referring to the artisanal fishermen’s knowledge of *S. guianensis* behaviour [22]. The questionnaire was approved by the Research Ethics Committee of the State University of Santa Cruz (*Comitê de Ética em Pesquisa da Universidade Estadual de Santa Cruz*: CEP-UESC) based on Resolution no. 196/1996 of the National Council of Health, which governs the ethical aspects of research involving humans. A free and informed consent form was read out and made available to those who participated in the study. The aims of the research were explained clearly at the beginning of each interview and those involved were asked whether they would like to participate in the study.

Interviews were recorded in writing and electronically on a digital recorder. They were then transcribed and quali-quantitatively analysed [23]. In addition, occasional boat expeditions were made with the fishermen to make direct comparisons between their reports and the observations made during the study by researchers. We thus sought to compare the fishermen’s verbal descriptions of the behaviour of *S. guianensis* with observations made by the researcher at the same time, in order to correctly interpret the verbal reports by interviewees.

Those interviewed were selected using the “snowball” method [24], first identifying and interviewing those fishermen who had been fishing for the longest time and who were considered knowledgeable by community members. These individuals, after being interviewed, indicated new potential informants for the research. A previous study conducted at the same location [25] facilitated the contact with the fishermen and access to information. Controls were performed through verification tests for consistency and validity of response [26], using repeated interviews in synchronic and diachronic situations. The consistency and robustness of the fishermen’s knowledge were assessed through prepared comparative cognition tables [23].

Ethnoecological data was analysed according to the model of integration of a range of individual skills [27]. For the purposes of comparison, consensual informant responses were obtained through the level of response fidelity [28] with the formula LF = (Ic/It) x 100, where LF = level of fidelity; Ic = number of informants who gave the consensual response (response most frequently cited); It = total number of informants who answered a specific question.

### Recording *S. guianensis* behaviour

The collection of *S. guianensis* behavioural data was conducted from October 2009 through September 2010, from a fixed observation point on the banks of the Pardo River estuary on a pier popularly known as Lloyd’s Bridge (15°40’59”S and 38°56’38”W). The pier extends 40 metres out over the river, is 30 metres wide, and is 11.5 metres high. Lloyd’s Bridge was chosen because it is an area frequently visited by *S. guianensis*. The bridge, being on average 5.5 m above the river, provides a good location for observing the dolphins. Also, it provides greater researcher safety compared to previously considered locations. In total, 609 hours of sampling was carried out over 93 days in alternating morning (08:00 to 13:00) and afternoon (13:00 to 18:00) observation sessions. Average observation time per month was 50.75 hours, with a minimum of 42 hours and a maximum of 60 hours.

Observations of behavioural activities were made using the “focal group sampling” method, where an individual is the focus of observation over a time period, although the same individual is not necessarily observed throughout the observation session [29]. Observations were conducted with the aid of binoculars (Bushnell 8 x 40) to follow animals that were distant from the observer (ca. 700 metres) and with the naked eye for those closeby. All incidences of individual behaviour on the surface which took place in the group were recorded continuously during the period the animals remained in the observation area (approximately 400 m^2^).

We also recorded the composition and size of the groups, the start and end time of the sighting, the animals’ direction and the amount of time they remained in the monitored area. All the information was noted on standardized field records and then transcribed onto electronic spreadsheets. Statistical validation was conducted using the Chi-squared test and Spearman’s rank correlation coefficient at a significance level of 0.05 [30]. The use of the term *in situ* in the text refers specifically to observations by the researchers.

## Results

Twenty-two (84.6%) of the fishermen interviewed are natives of the city of Canavieiras, three (11.5%) come from other municipalities in Bahia (Ilhéus, Caravelas and Valença), and only one fisherman comes from another state (Niterói, state of Rio de Janeiro). These four fishermen, although they were born in other cities, were identified as members of the community because they have lived for many years at this location. The interviewees’ principal age was from 31 to 50 years old (n = 12; 46%). Twenty-one interviewees (80.8%) said they had been working as fishermen for more than 20 years. The minimum recorded experience was 3 years and the maximum 53 years. Average time dedicated to fishing was 26.7 years. Only two fishermen (7.7%) had completed secondary school, while 22 (84.6%) had not completed primary school and two (7.7%) said that they had had no schooling, but could write their names. All the fishermen (n = 26) make use of canoes in their fishing activities. The fishermen cited four types of fishing equipment: line, net, cast net and rod. The fishermen mentioned line fishing most frequently (n = 16; 61.5%) and rod fishing least (n = 2; 7.7%).

*S. guianensis* were sighted in 38 of the 93 days of sampling (40.9%). A total of 70 groups and 252 individuals were observed. Of these, only two individuals were lone animals (0.79% of total individuals). They were present in the monitored area during 23 hours and 15 minutes, about 4% of sampling duration.

### General knowledge about *S. guianensis*

The Atalaia fishermen described the main biological and ecological aspects of the *S. guianensis* (Table 1). All the fishermen interviewed confirmed the presence of *S. guianensis* in the studied area. The consensual response of the fishermen (LF = 96%) regarding local nomenclature consisted of the word boto (a shortening of boto-cinza, the Brazilian Portuguese name for *S. guianensis*). According to the fishermen, botos are different from other species of the family Delphinidae because they swim up the Pardo River and the small tributaries in the region. The words dolphin and porpoise were exclusively used for marine Delphinidae. Only one of the interviewed fishermen used both the words boto and dolphin to designate *S. guianensis*. He stated that he has also seen the Pardo River species when fishing in open sea.

**Table 1:** **Atalaia fishermen's consensual responses about *S. guianensis***

**Aspect**	**N° of distinct responses**	**Consensual response**	**It**	**Ic**	**LF**
Presence of *S. guianensis* in the studied area	1	Yes	26	26	100
Local name for the species	2	Boto	26	25	96
Entry into /exit from the Pardo River	1	Yes	26	26	100
Reason for entering the Pardo River	3	‘To catch fish', to drink their blood	26	20	77
Time of the year they appear	2	The whole year	26	22	85
Differentiation	1	The younger ones are lighter; they get darker as they get older	26	26	100
Use of Pardo River	1	They use the inlet most; they go further in but they prefer the inlet	26	26	100
Presence of infants	2	Yes	26	21	81
Number of infants per group	3	One or two infants, depending on the size of the group	26	14	54
Time of year that infants are present	3	Throughout the year, sometimes there are more but I'm not sure exactly	26	13	50
Time of day that the dolphins appear	2	It depends on the tide	26	19	73
Gregarious behaviour	1	They live in groups. Lone individuals are rarely seen.	26	26	100
Number of individuals per group	1	From two to eight	26	26	100
Main observed activity	2	‘Catching fishing' and ‘passing normally'	26	16	61
Feeding strategy	1	Circling and chasing rake stardrum (*Stellifer rastrifer)*	26	26	100
Noticeable behaviour	5	Beating the tail on the branches of the mangrove to scare the fish	20	10	50
Surface behaviour	3	Criss-crossing each other. Lone jumps. Jumps and rotates, executing a 'pirouette'	26	18	69
Parts of the body frequently exposed	3	The 'beak', the 'wings', the tail	26	19	73
Reproductive behaviour	1	No	26	26	100
Play	1	Jumps, crosses, slaps wing, somersaults, belly jumps, back jumps, raises its head out of the water then sinks back, throws the rake stardrum (*Stellifer rastrifer)* into the air	26	26	100
Interaction with fishing	1	Helps the fisherman. The fish flee to shallow areas	26	26	100
Interaction with boats	1	Are used to them but scared when there is a lot of noise	26	26	100
Interaction with fishing gear	2	Rarely become entangled. They see the nets	26	23	88

It = number of informants who responded; Ic = number of informants who gave the consensual response; LF = level of fidelity.

All the fishermen confirmed that the species regularly moves between the estuary region and the open sea. The main reason for *S. guianensis* to regularly enter and exit the Pardo River estuary is associated with food. Twenty fishermen (LF = 77%) stated that the dolphins enter the estuary region in search for fish blood. In other words, these fishermen believed that the dolphins do not consume totally their prey but merely “suck” their blood. Two fishermen said that they had heard about this feeding habit but did not really believe that it happened. The other fishermen (n = 4) did not mention the issue of blood “sucking”.

All the fishermen reported that it is possible to differentiate the animals’ age based on their colouration patterns. According to them, the younger individuals are light grey and/or pink, while the adults are predominantly grey, with only the ventral area being lighter. The fishermen informed us that *S. guianensis* uses preferentially the estuary mouth and the middle section of the Pardo River where sea water is mixed with freshwater. The species was also observed in the upper section where the tides still influence water levels, but where the water is predominantly fresh.

The interviewees reported a positive relationship between themselves and *S. guianensis* in terms of fishing, since the dolphins tend to herd shoals of fish into shallower areas, facilitating the fishermen’s capture of fish. Furthermore, they mentioned that most dolphins are accustomed to the boat traffic, although the dolphins rarely approach the fishermen. Accidental entanglement in gill nets was said to be infrequent, likely due to the animals’ visual acuity (LF = 88%).

### Ethnoecological knowledge about *S. guianensis*

The Atalaia fishermen described some behaviour aspects of *S. guianensis* (Table 2). All the fishermen interviewed noted *S. guianensis* presence in the Pardo River estuary throughout the year. According to them, the species is typically gregarious, characterized by groups of two to eight individuals, while lone animals are seldom observed.

**Table 2:** **Table of comparative cognition between the ethological observations made *in situ *and the Atalaia fishermen's knowledge regarding *S. guianensis *behaviour, including their respective fidelity levels **

**Behaviour**	*** In situ *observation**	**Fisherman’s citation**	**Level of fidelity**
Seasonality	*S. guianensis* groups were observed in each of the 12 months of observation	“We see them throughout the year” (26)	1
Movement and tidal cycle	Individuals entered more frequently at high tide and exited more frequently at low tide, following the movement of the tide	“It depends on the tide. But they enter more frequently at high tide and exit more frequently at low tide. They are smart fish" (19)	0.73
Gregarious	A total of 70 groups and 252 individuals were recorded of which only two were lone animals	“They only travel in groups. It's unusual to see them alone" (26)	1
	The size of groups ranged from two to seven individuals	“There are groups of two, four, six, eight" (26)	1
Feeding	Feeding events were the behaviours most frequent recorded	“They come here to fish” (26)	1
	Circling and chasing were most frequently observed	“They create a circle. They chase the rake stardrum (*Stellifer rastrifer)*from underneath and all the fish jump to get away” (26)	1
	An adult beats its fin on mangrove roots and three other individuals then execute shallow dives	“They beat their tails on the mangrove branches to scare the fish” (10)	0.38
	*	“They go to the shallows, right up to the water's edge, to get the beached fish. Then they go back into the water” (3)	*
Reproductive	Positive correlation between number of infants and size of group	“We always see some little ones with them. One, two, it depends on the size of the group” (14)	0.53
	Infants were not seen in November	“They have infants with them all year” (17)	0.65
	Significant difference in the number of infants over the 12 months of observation	"There is a time of year when there are more, but I'm not sure when" (13)	0.50
	No reproductive events were recorded	“I've never seen them mating around here" (26)	1
On the surface	Leaping, slapping and spyhopping were recorded	“Criss-crossing each other. Lone jumps. Jumps and rotates, performing a "pirouette" (18) "Shows its beak, slaps its wing and tail" (19)	0.69
			0.73
Play	Locomotor, social and play with object were recorded	“Jumps, crosses, slaps wing, somersaults, jumps, jumps onto mother, back flips, raises its head out of the water then sinks back, throws rake stardrum (*Stellifer rastrifer)*into the area. All kinds of play." (26)	1

Number of citations is in brackets.

*Behaviour not observed *in situ* and with no equivalence for the *S. guianensis* species in the literature.

From the *in situ* observations, the *S. guianensis* presence was recorded throughout the 12 months of monitoring. Group size varied from two to seven individuals, with groups of two, three or four individuals being the most frequent. Lone animals were sighted on only two occasions (0.79% of the total number of individuals).

One of the fishermen’s consensual responses indicated the existence of a relationship between *S. guianensis* movements in the estuary and the cycle of the tides. The dolphins most often enter the estuary region during high tide, taking advantage of the displacement of water to move into the estuary and they most often exit it as the tide goes out. Although the inverse movement was also mentioned, the fishermen asserted that the former is more frequently observed.

**Figure 1:**
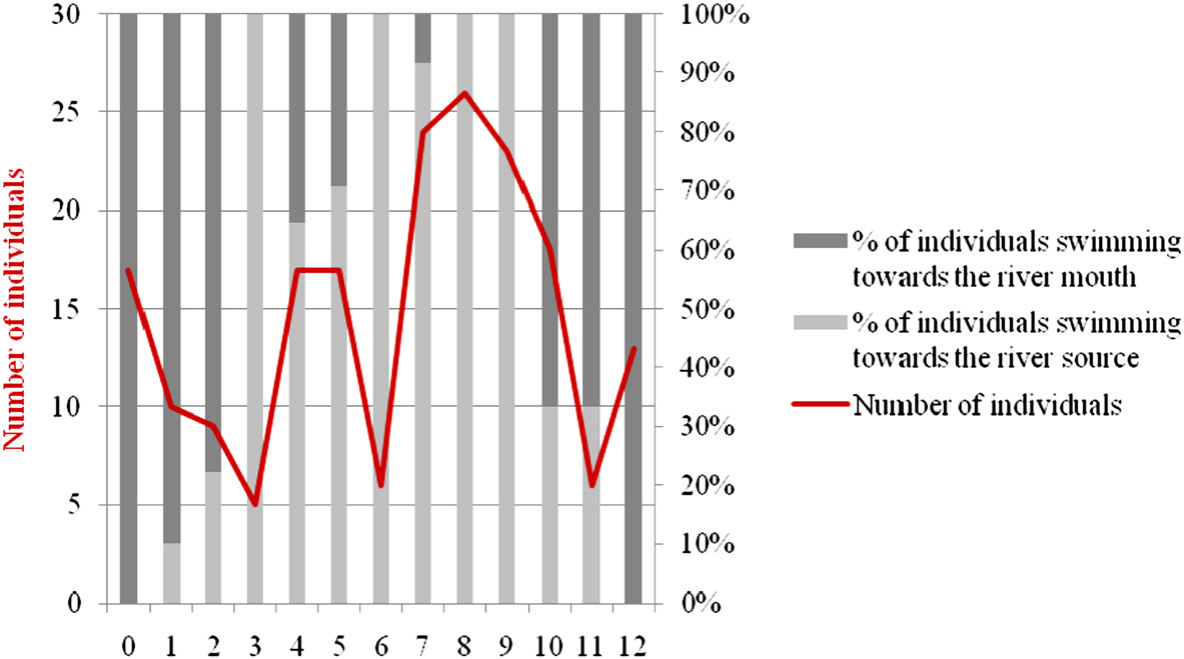
**Swimming direction of* S. guianensis *individuals within the monitored area according to the tide, between October 2009 and September 2010 (numbers indicate the hours elapsed since the last tide: "0" is low tide, "6" is high tide).**

*In situ* observations indicated that the total number of individuals moving towards the river mouth or towards the river source (n = 191, Figure 1) varied significantly with the state of the tide (Chi-square goodness-of fit test, X² = 41.71; df = 12; p < 0.001). Individuals were more likely to enter at high tide and to exit at low tide, following the displacement of the tide.

**Figure 2:**
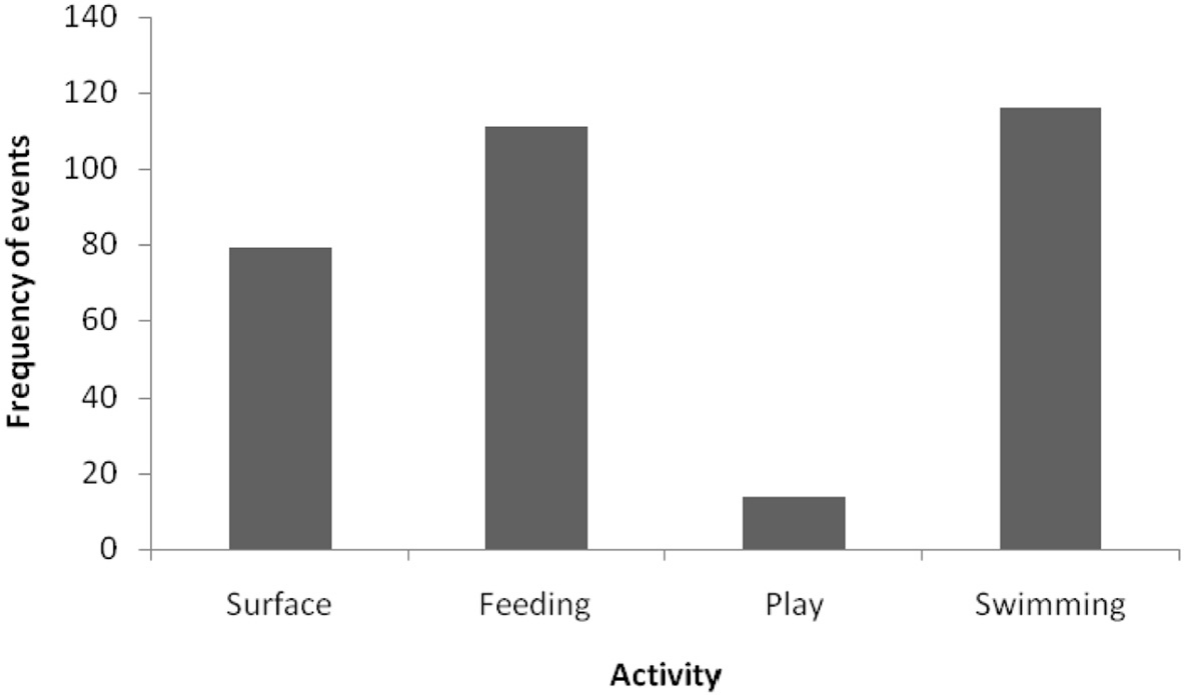
**Frequency of* S. guianensis *behavioural events in Canavieiras between October 2009 and September 2010 (n = 320).**

All the fishermen associated the presence of *S. guianensis* in the Pardo River estuary with the search for food. Movement and feeding were the activities most frequently recorded during observations from the fixed point (Figure 2).

The fishermen described two principal feeding strategies: “circling” and “chasing”. The first occurs when circling individuals work cooperatively in a group to apprehend prey within a defined area. Chasing consists of individuals moving towards the prey either slowly or quickly; the prey then jumps to escape the attack.

The feeding behaviours observed *in situ* were: (i) herding towards an obstacle [31], (ii) catching by moving forwards [32], (iii) catching without jumping [33], (iv) consuming prey [34], (v) forming a circle [31], (vi) attacking with dorsal upright [33], (vii) cooperative hunting [35], (viii) coordinated hunting [36], (ix) criss-cross fishing [31], (x) individual fishing [35], (xi) a sequence of deep dives [31], and (xii) infants fishing (following and training) [31].

All events that indicated any type of cooperative organization between the individuals in a group (e.g. herding towards an obstacle, forming a circle) we grouped into the ethnocategory “circling”. The behaviours that involved chasing prey in flight with leaping (e.g. catching by moving forwards, chasing with dorsal upright) were brought together under the ethnocategory “chasing”. Three events observed *in situ* (individual fishing, infants fishing and a sequence of deep dives) did not fit into either of the two ethnocategories and were therefore grouped into the “others” category. The frequency of the events of the two ethnocategories “circling” and “chasing” was higher than the “other” category.

Three fishermen interviewed described a feeding behaviour that is not described in the *S. guianensis* literature. According to them, the dolphins intentionally beach onto the sandbanks and riverbanks of the Pardo River estuary in order to catch prey heading into shallower waters. By doing this, the dolphins exhibit their bodies partially or completely out of the water, and then return to water. However, this behaviour was not observed *in situ*.

Another feeding behaviour unreported in scientific literature was described by ten fishermen: the animal beat the caudal fin against the mangrove roots in order to disperse prey hidden within. According to the fishermen, this behaviour usually takes place at night, although it may occur in the day. We observed this behaviour *in situ* on one occasion, during the day. One adult beat its caudal fin on mangrove roots and the other three individuals of the group – two adults and one infant – then executed shallow dives in the vicinity. Upon questioning, the fishermen confirmed that our description corresponded to the behaviour they had observed.

**Figure 3:**
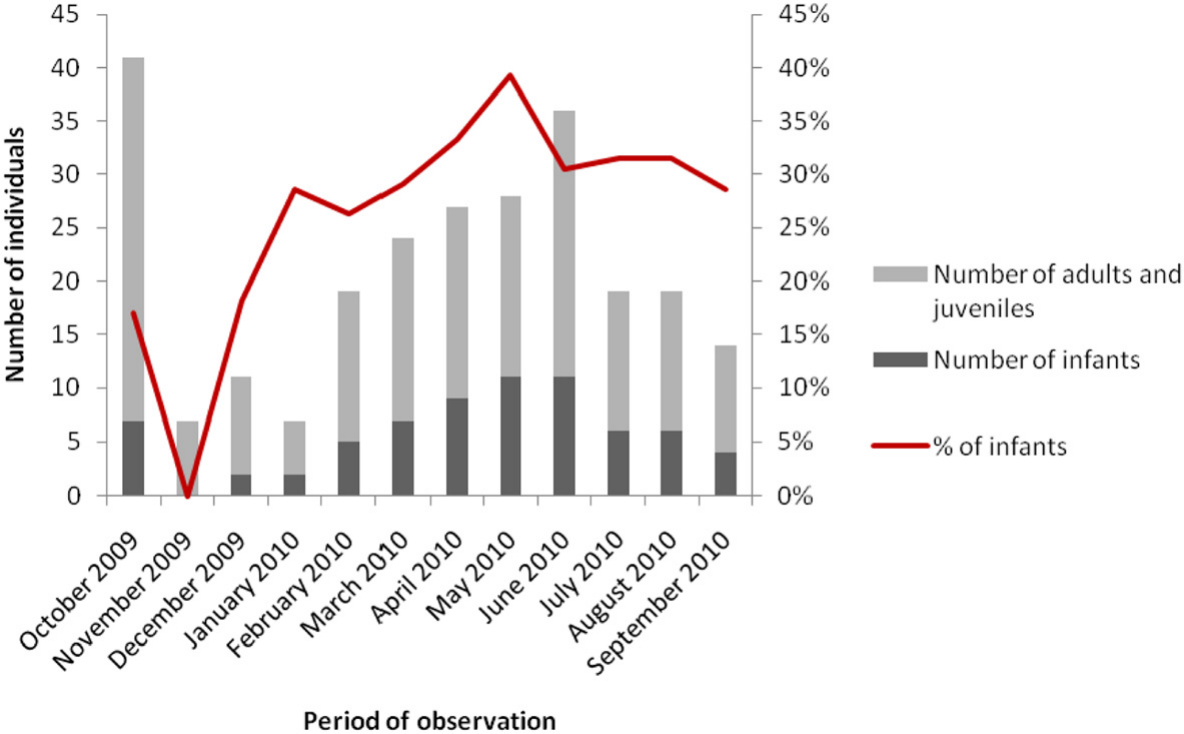
**Number of individuals by age group and percentage of* S. guianensis *infants observed in Canavieiras between October 2009 and September 2010.**

The fishermen reported that infants are present throughout the year and that the number of infants in a group is proportional to its size. According to responses by fishermen, the infants are more frequently sighted during specific periods, although they did not know when the greatest number of births occurs. Infants were recorded in eleven of the twelve months of monitoring (except in November). May was the month in which we recorded the highest percentage of infants (Figure 3). There was a positive and significant correlation (r_s_ = 0.870; p = 0.024) between the number of infants and the size of the group.

All the fishermen interviewed reported that they had never witnessed sexual behaviour in this species in the region. No events associated with reproduction, such as copulation for example, were recorded *in situ*.

In regards to surface behaviour, the fishermen reported that *S. guianensis* usually shows the rostrum, performs leaps and slaps its head and tail. They also mentioned a behaviour locally known as “pirouette” which basically consists of a leap and a turn. We recorded nine surface behaviours: slapping the tail, slapping with head dorsal side and the reverse, spyhopping, total leap, partial leap and crossed leap [35], pectoral aerial behaviour [36] and aerial rolling [33]. The leaps, slaps and spyhopping were the behaviours most frequent in our observations.

A leap similar to the one described as a pirouette by the fishermen was observed on one occasion. The individual performed a leap of total body exposure, turning on its own axis (a turn of 360°). Similar leap was recorded in Baía Formosa, state of Rio Grande do Norte, Brazil [37].

When referring to their observations of *S. guianensis* play, the fishermen mentioned leaps, head and tail slaps, interaction with objects and spyhopping. Three types of play were recorded during the *in situ* observations: (i) locomotor play (ii) social play and (iii) play with object [38].

## Discussion

*S. guianensis* is a typically coastal species known to move regularly between bays, estuaries, areas close to mangrove and the open sea [33,39]. This corroborates the information given by the Atalaia fishermen when they confirmed that the species regularly moves between the Pardo River estuary and the open sea.

The feeding habit described by the fishermen as “sucking” the blood of their prey without necessarily consuming it whole was also reported to Zappes et al. [12] in a study carried out in fishing communities in the states of Bahia, Espírito Santo, Rio de Janeiro and São Paulo. The main explanation provided by the fishermen interviewed in Atalaia is that they frequently see dead fish, whose bodies are covered with teeth marks, floating in the river. This is the same justification given by the fishermen interviewed by Zappes et al. [12]. A further explanation given by the Atalaia fishermen is that they often see *S. guianensis* holding prey in its mouth, corresponding to the moment when the dolphins “suck” their prey's blood.

During the observations, dead fish were sighted in the Pardo River. One possible explanation is that these fish are the result of clashes between *S. guianensis* and its prey during foraging, such as in the "consuming prey" behaviour described by Nascimento et al. [34]. In this behaviour, the prey is thrown into the air or against the surface of the water following capture and then may, or may not, be recaptured. Perhaps the fish found by the fishermen are indeed prey captured by the dolphins but not consumed.

Another explanation might be that the fishermen are interpreting behaviour exhibited by *S. guianensis*, such as the "catch" [33], based on their own perceptions. During the catch, the dolphin emerges with the prey in its mouth and perhaps this behaviour leads the fisherman to believe that the animals “suck” the blood of their prey.

A third explanation given by the interviewees is based on the fact that the musculature of *S. guianensis* contains a lot of blood. According to the fishermen, this characteristic results from their habit of “sucking” the blood of their prey. To summarise, the skeletal muscles of animals such as dolphins are rich in fast-twitch fibres. These fibres have a dense blood supply because they need to provide the oxygen required for active locomotion over long periods [40].

The fishermen who said they knew of the “sucking” prey habit but did not believe in it explained that, in fact, it was a story made up by the older members of the community. This would prevent children from having accidents by going near or into the Pardo River.

The *S. guianensis* age differentiation made by the fishermen based on colouration patterns is compatible with the scientific literature. According to Hetzel and Lodi [39] and Flores and Silva [9], the colouration of dorsal area and fins is usually grey, while the ventral area is lighter. This colouration pattern changes in line with the animal's age. The younger individuals' colouration varies from pink to light grey, just as the Atalaia fishermen reported.

The fishermen's reporting of the preferential use of the lower and middle sections of the Pardo River estuary rather than to the areas further upstream could not be tested, since the *in situ* observations were restricted to a single point of observation on the estuary banks. However, an ecological study of *S. guianensis* conducted in the same region [41] confirmed this information.

The positive fishing relationship between *S. guianensis* and the Atalaia fishermen was also recorded by Przbylski and Monteiro-Filho [42] in three locations on the Parana coast. According to the fishermen, *S. guianensis* usually herd shoals towards shallower areas, thus facilitating the fishermen's capture of the fish.

The possible habituation of *S. guianensis* relating to boat traffic, reported by the Atalaia fishermen, was also observed by Santos [43] in Ilhéus, in the state of Bahia, and by Araújo et al. [44] in the state of Pernambuco. During observations of encounters between *S. guianensis* and boats, neutral reactions were predominant, independent of the type of craft.

Accidental entanglement in nets was rarely observed by the Atalaia fishermen and is compatible with Zappes' statement [45] that, on a local scale, the rate of accidental capture of *S. guianensis* in passive fishing gear, such as gill nets, is low. However, given that we are dealing with a fishing modality expressive in over 9,000 km of Brazilian coast, the sum of local accidents may represent a threat to *S. guianensis* populations [46].

The year-long presence of *S. guianensis* in the Pardo River estuary was also recorded by Lodi [47] in the Bay of Paraty (RJ), Geise et al. [48] in the estuarine-lake complex of Cananéia (SP) and Izidoro [49] in the Port of Ilhéus (BA). In the study of Zappes et al. [12], artisanal fishermen also reported sightings of dolphins throughout the year. According to Ballance [50], loyalty to the place is directly related to the supply of food resources. The residence of *S. guianensis* during the whole of the year is an indication that the area possesses sufficient resources to sustain the population throughout this period [51].

The relationship between *S. guianensis* movements and the tidal cycle differed from some studies conducted in other regions of Brazil [17,52,53]. One possible explanation is that during flood and high tides the animals follow the movement of shoals that use the current to enter the Pardo River estuary. According to Bordino et al. [54], Cremer et al. [55] and Würsig and Würsig [56], a number of fish species use the movement of the tides to enter systems such as estuaries. During ebb and low tides, the dolphins probably move to areas that are even shallower, compared to the area studied. At low tide these locations facilitate the search for food, since prey is concentrated in a small volume of water. Another explanation is that the animals make use of the current to enter and exit the estuary without expending much energy, which would otherwise be required for movements against the current. The Atalaia fishermen used the latter explanation to justify this pattern of movement.

Given the *in situ* observations and taking into consideration the Atalaia fishermen's reports, we can assert that events associated with feeding are one of the principal activities exhibited by the *S. guianensis* species in the River Pardo estuary. In the study of Zappes et al. [12], the fishermen also mentioned foraging as the most frequently observed activity. A number of studies describe feeding as the principal activity of *S. guianensis* within their area of study [46,53,57,58].

Intentional beaching, described by the Atalaia fishermen in relation to the *S. guianensis* species and not confirmed *in situ*, is a hunting method only recorded in the scientific literature for groups of *Orcinus orca* in Argentina and for *Tursiops truncatus* in certain locations in the southern United States [39]. However, Santos [59] recently suggested including *S. guianensis* in the list of cetaceans performing beach hunting since they frequently catch preys near exposed sand.

Other feeding behaviour described by the Atalaia fishermen that has no equivalence in the scientific literature was confirmed *in situ*. This behaviour consisted of beating the caudal fin against mangrove roots in order to disperse the prey hidden within. This is the first report of such behaviour for this species. This fact illustrates the value of local ethnozoological knowledge for academic information in supplying complementary data about the biology and ecology of animal species, as well as supporting the formulation of scientific hypothesis [8]. There are other examples in the literature that corroborate this assertion, such as Marques' [26] study, which developed an apparently implausible hypothesis based on information provided by fishermen about an important food item for the catfish *Arius herzbergii*. Testing the fishermen's statements in scientific research revealed that the catfish consumed terrestrial insects (Ephemeroptera), a trophic relationship unknown within the academic context until that point.

The fishermen reported observing infants throughout the year, and the *in situ* observations recorded infants for all months but not in November 2009. Gonçalves [60] and Reis [35] also observed infants in the Port of Ilhéus (BA) in every month during which data were collected with just one exception (February). Some studies have indicated the presence of infants in every month of the year, without exception [17,47,61]. From November 2009 to January 2010, the number of dolphins observed in the Pardo River estuary was less than in the other months of observation. Therefore, the probability of not observing infants was higher. A diminution in the availability of food resources during this period may force the animals to move to other areas where food was more abundant, as suggested for other populations [47].

Although the fishermen did not specify periods in which they saw infants more frequently, our observations indicate May 2010 to be the period of highest sightings. In other regions [47,48,52,62], an increase in the number of infants occurred during the Southern Hemisphere summer (December to February). The increase observed in the birth rate may be an adaptive response to a seasonal variation in prey availability given that lactating females supplied energy favours the survival of their infants. The fishermen interviewed by Zappes et al. [12] also cited the presence of infants throughout the whole year and said they did not know of a period of greater frequency.

The number of infants in each group was directly proportional to the size of the groups, a result similar to that obtained by Lodi [47] in the Bay of Paraty, Rio de Janeiro, as reported by the Atalaia fishermen. The underwater sexual behaviour of *S. guianensis* and its eventual occurrence in deeper or open waters can explain the absence of records of sexual events in the interviewed fishermen’s reports and the *in situ* observations [34].

The most frequent surface behaviour observed *in situ* coincided with the behaviour described by the Atalaia fishermen, confirming the compatibility between the two sources of information. The leap that the Atalaia fishermen called pirouette is characteristic of *Stenella longirostris* which executes a total leap rotating various times around the axis of its body before returning to the water [39]. Unlike *S. longirostrisS. guianensis* performed only one complete rotation around its own axis. The pirouette also differs from the “somersault” described by Nascimento et al. [34] for *S. guianensis*, where the leap involves a turning the body over the head (with the caudal fin describing a circumference around the head) and not one which rotates around the body’s axis.

The same surface behaviour descriptions are repeated when the fishermen describe play behaviour. The fishermen mentioned physical contact between infants and adults and *S. guianensis* interacting with its prey by throwing it into the air. All these behaviours were observed *in situ* and were considered to be play, with the exception of the last, which was considered to be a feeding behaviour (“consuming prey” [34]). The play cited by the Atalaia fishermen was also that most frequently observed by Spinelli et al. [38] on Pipa Beach (RN). Play with objects was also recorded in the Pardo River estuary. Play is recognized in many mammal species, is predominantly carried out by infants and is beneficial to the individuals involved by preparing them for possible challenges (e.g. motor, emotional) throughout their development [38].

## Conclusions

The knowledge presented by the Atalaia fishermen was a valid and useful source of information for the behavioural study of *S. guianensis* in Canavieiras. They have demonstrated a body of knowledge about the natural history and behaviour of *S. guianensis* which describes in detail the main behavioural aspects of the species. Most of the behaviours described were compatible with the observations conducted *in situ* and with data found in the scientific literature. One of the behaviours mentioned by the fishermen and that did not have equivalence in the *S. guianensis* literature was confirmed *in situ* and, therefore, constitutes the first official record for this species. Artisanal fishermen in different regions in Brazil share certain cultural knowledge. The information obtained through this study may support future initiatives aimed at the conservation of *S. guianensis* in Canavieiras, which has an important role in the culture of local fishing communities.

## Competing interests

The authors declare that they have no competing interests.

## Authors’ contributions

MEBC carried out the field research and drafted the manuscript. YLP and EMCN participated in its design and coordination, and helped to draft the manuscript. All authors read and approved the final manuscript.
